# Assessment of Posterior Pharyngeal Airway Changes After Orthognathic Surgery Using Barium Sulfate

**DOI:** 10.7759/cureus.42836

**Published:** 2023-08-02

**Authors:** Jyoti M Biradar, Mahesh Kumar, Srinath N, Harshawardhan Ravindra Kadam, Abdullah N Tamboli, Swapnil U Shinde

**Affiliations:** 1 Department of Oral and Maxillofacial Surgery, Bharati Vidyapeeth (Deemed to be University) Dental College and Hospital, Sangli, IND; 2 Department of Oral and Maxillofacial Surgery, Sri Siddhartha Academy of Higher Education, Tumkur, IND; 3 Department of Oral and Maxillofacial Surgery, Krishnadevaraya College of Dental Sciences, Bangalore, IND

**Keywords:** barium sulphate, orthognathic surgery, hyoid bone, obstructive sleep apnea, posterior airway space

## Abstract

Introduction: Obstructive sleep apnea (OSA), caused by airway narrowing, is likely to occur if the mandibular plane to hyoid distance is greater than 15.4 mm and the posterior airway space (PAS) is less than 11 mm. OSA may be caused by mandibular deficit, bimaxillary retrusion, increased lower facial height, extended soft palate, a large tongue base, and a posteroinferiorly positioned hyoid bone. Snoring and drowsiness during exercise are symptoms of OSA, which is a risk factor for high blood pressure, heart disease, and stroke, and these can result in car crashes. However, orthognathic surgery can improve dental occlusion and aesthetics by adjusting facial bone position, shape, and size. When bones move, the position and tension of soft tissues change. These novel soft tissue interactions, especially when anteroposterior, change the face’s appearance and PAS dimensions. This study uses barium sulfate paste to enhance lateral cephalograms before and after orthognathic surgery to assess posterior pharyngeal airway changes.

Materials and methods: Barium sulfate was mixed with water to make a paste for the tongue's dorsum. A preoperative digital lateral cephalogram was obtained, and a postoperative evaluation was conducted six weeks after the procedure. In the cephalostat, the Frankfort horizontal and median planes were aligned parallel to the floor, and a radiograph was taken after the breathing cycle to standardize the hyoid bone location. Preoperative lateral cephalogram analysis using Burstone's hard tissue landmarks confirmed skeletal class II or III deformities. First, the narrowest part of the posterior pharyngeal airway was measured. Second, the narrowest portion between the soft palate and posterior pharyngeal wall parallel to the Frankfort horizontal plane was measured preoperatively, and the procedure was repeated six weeks postop.

Results: Complexity characterizes the pharyngeal airway, which, along with the surrounding structures, facilitates the bodily functions of eating, talking, and breathing. The pharyngeal airway is located behind the nose, mouth, and larynx, and adjusting the jaws changes the size and structure of the pharyngeal airway and surrounding soft tissues, which may affect breathing. A statistically significant change is detected in the posterior palatal and posterior lingual airways after different orthognathic operations. After the mandible is moved forward, both the posterior palatal and posterior lingual airways enlarge. Furthermore, the soft palate exhibits slight decreases in length, thickness, and angle. Additionally, there is an anterosuperior displacement of the hyoid bone. Following maxillary superior impaction, mandibular autorotation is seen in a counterclockwise direction, which has the same result as that of mandibular advancement.

Conclusion: It is essential to consider these soft tissue changes when planning orthognathic procedures, as alterations in the pharyngeal airway may impact the patient's postoperative breathing and overall health. Patients with OSA or those at risk of developing it should be closely evaluated and managed appropriately during the surgical planning process.

## Introduction

The posterior pharyngeal airway is the upper respiratory passage composed of the nasopharynx, oropharynx, and hypopharynx. It is widest at the nasopharynx, narrow at the oropharynx, and narrowest at the hypopharynx [1]. The size of the soft palate, tongue, and hyoid bone affects the size of the posterior airway. Being connected either directly or indirectly to the maxilla and mandible, their motions will affect the size of the mouth cavity and the pharyngeal airway [2].

Narrowing of the airway is associated with sleep-related breathing disorders, such as obstructive sleep apnea (OSA). Posterior airway space (PAS) of less than 11 mm and mandibular plane to a hyoid distance of more than 15.4 mm is indicative of OSA [2]. The mandibular deficit, bimaxillary retrusion, increased lower facial height, extended soft palate, a big base of the tongue, and a posteroinferiorly positioned hyoid bone are only a few of the morphological and physiological variables that have been proposed as causes of OSA [3]. Common symptoms include drowsiness during physical activity and snoring. OSA is known to cause automobile crashes and is a risk factor for high blood pressure, heart disease, and stroke [3,4].

By adjusting the position, form, and size of the facial bones, orthognathic surgery may improve dental occlusion and aesthetics. When bones shift, the soft tissues connected to them shift position, which results in changes in tension. Significant alterations in face appearance and PAS dimensions are brought about by these novel soft tissue interactions, particularly when an anteroposterior component is present [2,5]. Isolated mandibular setback treatments have been linked by many researchers to the development of sleep-related respiratory difficulties [3]. Postoperatively, studies have demonstrated that the retrolingual and hypopharyngeal airways narrow and that the hyoid bone and tongue are displaced posteroinferiorly. However, maxillomandibular advancement has been shown to have a positive effect on respiratory issues. By moving the bony connections of the suprahyoid and velopharyngeal muscles and tendons forward, the PAS is enlarged [5]. However, an orthognathic operation may cause permanent and detrimental changes to the jaws and PAS, which may cause or exacerbate a breathing condition. On the other hand, there are various orthognathic treatments that could really improve breathing [3]. The pharynx, dentofacial, and craniofacial structures all interact with one another due to their close proximity [6].

As part of our research, we analyzed the before-and-after pharyngeal airway dimensions of patients who had undergone orthognathic surgery to see whether they had decreased or increased by applying a layer of barium sulfate paste as a radiopaque contrast agent on the tongue. Preoperative and postoperative digitalized lateral cephalometric radiographs were taken, with an approximate interval of six weeks between them, to assess changes in skeletal characteristics and the oropharyngeal airway [3].

## Materials and methods

Source of data

Patients who sought care at the orthodontics and oral and maxillofacial surgery clinics at Bangalore's Krishnadevaraya College of Dental Sciences participated in the research. Ethical clearance was obtained from the institutional ethical committee with the institutional review board number - IEC/OS/08/11/2013.

Data collection method

**Figure 1:**
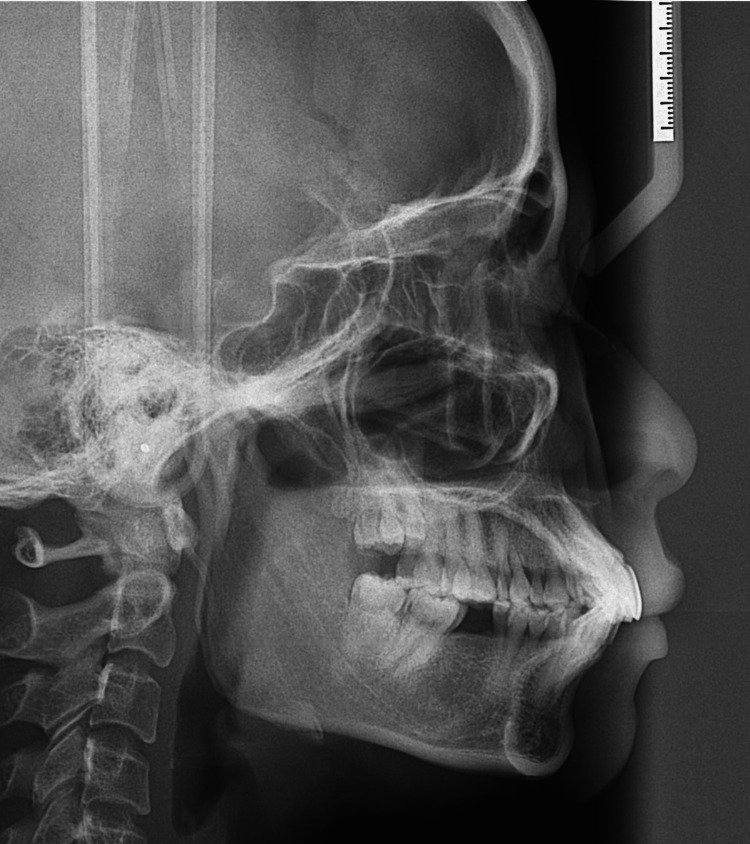
Lateral cephalogram

A minimum sample size of 10 patients was determined for this study. These patients had undergone orthognathic surgery for the correction of various maxillomandibular deformities. Preoperative and approximately six-week postoperative digitalized lateral cephalometric radiographs were used to evaluate the patient's skeletal features and pharyngeal airway (Figure 1).

The soft tissue identity of the tongue was enhanced by painting a coating of barium sulfate paste over the dorsum of the tongue. The hyoid bone was positioned consistently in the radiograph by exposing it at the conclusion of the patient's expiration. The head was radiographed in a cephalometric fashion. A bony and pharyngeal soft-tissue apex was found. Class III and Class II skeletal malocclusion patients were included in the research. Patients excluded were medically compromised patients not considered fit for surgery and those with an allergy to barium sulfate.

Procedure

**Figure 2:**
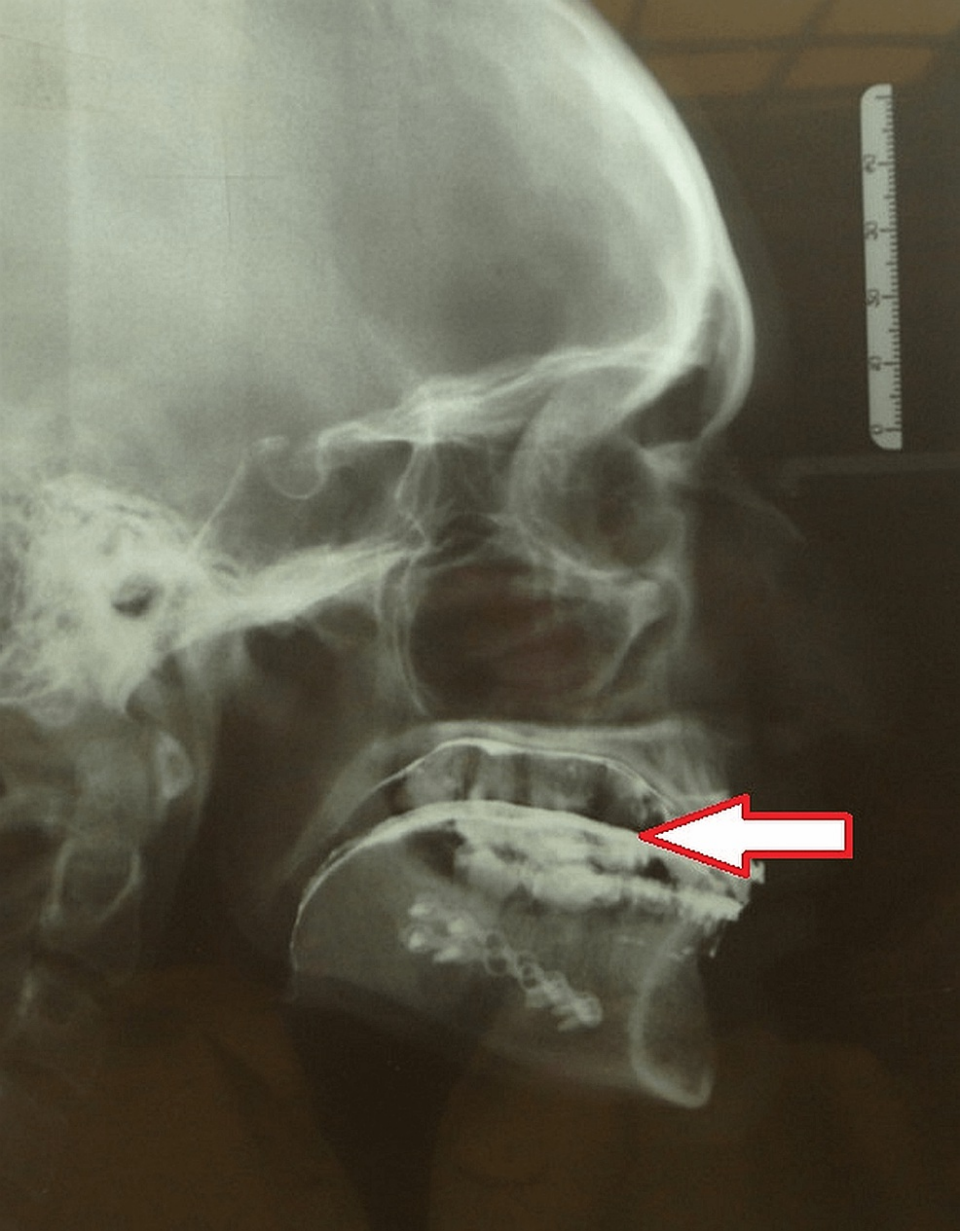
Post-operative cephalogram after barium application Arrow:  the barium marking on the cephalogram

A homogeneous paste of barium sulphate was prepared by mixing it with water and painted on the dorsum of the tongue. A digital lateral cephalogram was taken both preoperatively and six weeks post-operation. Both the Frankfort horizontal plane and the median plane were parallel to the floor when the individuals were positioned in the cephalostat. The hyoid bone located in the radiograph was standardized by taking it at the conclusion of the breathing cycle (Figure 2).

A preoperative lateral cephalogram was analyzed using the hard tissue landmarks stated in Burstone’s analysis for a confirmatory diagnosis of skeletal class II or III deformities. The width of the posterior pharyngeal airway was measured at two sites. First, a measurement was taken at the smallest opening between the base of the tongue and the back of the throat. Second, a measurement was taken before surgery at the point of minimal space between the soft palate and the posterior pharyngeal wall. The procedure was repeated six weeks after surgery.

**Figure 3:**
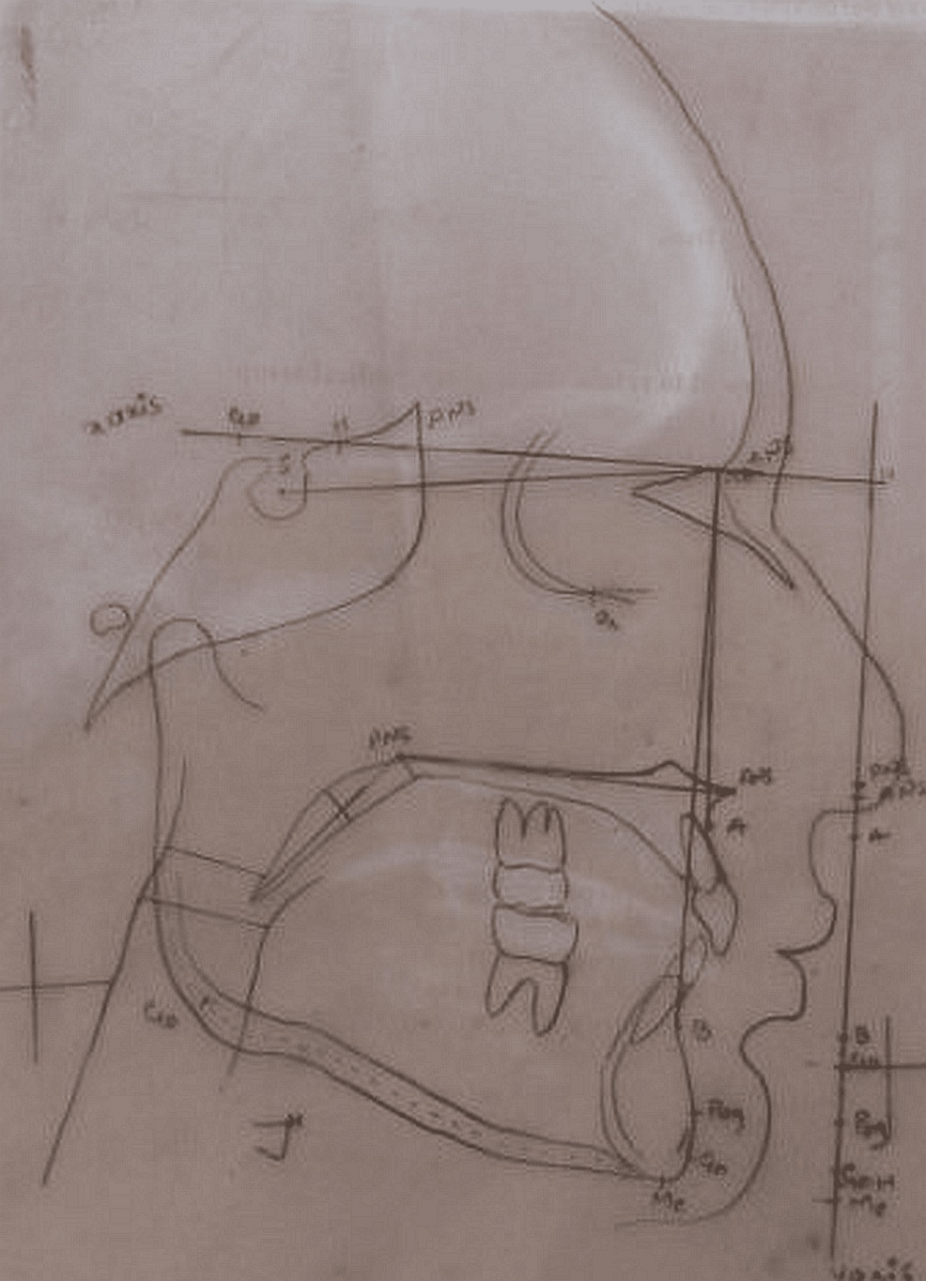
Tracing done for the analysis

The radiographic tracing was done on 0.003-inch acetate paper using a 0.5-mm lead pencil, a ruler, and diagonals. All tracing was done by a single investigator. The measurement was calculated based on the anatomical landmarks (Figure 3).

Details of the measurements used as shown in Table 1.

**Table 1:** Details of the measurements used

Measurement	
Upper anterior facial height (UAFH)	The distance from the top of the nose (nasion) to the top of the nose (anterior nasal spine).
Lower anterior facial height (LAFH)	The distance from the tip of the anterior nasal spine to the cranial bone of Menton.
Lower posterior facial height (LPFH)	The distance from the posterior nasal spine (PNS) to the gonion (Go).
Hyoid-ANS	The anteroposterior movement of the hyoid bone in millimeters.
Hyoid-gonion	The superoinferior movement of the hyoid bone in millimeters.
Soft palate length	The distance from the back of the nose to the front of the palate in millimeters.
Soft palate thickness	Points on the nasal and oral surfaces of the soft palate are used to measure the thickness of the soft palate in millimeters.
Soft palate angle	Concave palatal length of the soft palate and angle at which it meets the maxillary plane.
Posterior palatal airway	The distance from the posterior pharyngeal wall to a location on the soft palate.
Posterior lingual airway	Distance (in millimeters) between the back of the tongue and the pharynx at its narrowest point.

Statistical analysis

The data was collected and compiled in a Microsoft Office Excel sheet for statistical analysis. SPSS V17.0 (IBM Corp., Armonk, NY) was used to analyze the data. A non-parametric test was used for comparison of preoperative and postoperative values, and a Wilcoxon signed-rank test was conducted.

## Results

This is an in vivo study consisting of 10 patients, of whom five were females and five were males. All patients were treated surgically for various maxilla mandibular discrepancies. Out of the 10 patients, four underwent mandibular advancement, four underwent maxillary superior impaction, one underwent mandibular setback, and one underwent surgically assisted maxillary expansion.

Since different surgical procedures were performed on different patients, preoperative and postoperative craniofacial and oropharyngeal dimensions were measured and recorded. A statistical analysis was conducted using the Wilcoxon signed-rank test, and the readings were tabulated. Craniofacial dimensions as shown in Table 2.

**Table 2:** Craniofacial dimensions SNA: Sella, nasion, A point, SNB: Sella, nasion, B point, ANB: Relative anteroposterior position between the maxilla and mandible, UAFH: Distance from the top of the nose (nasion) to the top of the nose (anterior nasal spine), LAFH: Face length from the tip of the anterior nasal spine to the cranial bone of Menton, LPFH: Lower posterior facial height, H-ANS: Antero posterior movement of hyoid bone, H-GO: Distance from hyoid bone to gonion

Variables	Number of patients	Preoperative, mean±sd	Postoperative, mean±sd	Mean difference	Mean difference in %	P-value
SNA	10	78.70±4.191	79.10±3.178	0.4	0.5056	0.765
SNB	10	77.00±6.272	78.50±2.677	1.5	1.91	0.355
ANB	10	1.70±5.056	0.80±1.814	-0.9	-112.5	0.212
UAFH	10	49.80±6.143	50.10±7.475	0.3	0.598	0.953
LAFH	10	72.10±10.939	72.90±10.181	0.8	1.097	0.634
LPFH	10	42.10±7.680	42.950±7.9458	0.85	1.979	0.383
H-ANS	10	54.050±11.6439	53.70±8.301	-0.35	-0.651	0.505
H-GO	10	26.70±11.528	24.150±12.1519	-2.55	-10.55	0.096

The SNA mean difference is 0.4, the percentage mean difference is 0.505, and the P-value is 0.765, which is statistically not significant. The SNB mean difference is 1.5, the percentage mean difference is 1.91, and the P-value is 0.355, which is statistically not significant. The UAFH mean difference is -0.9, the percentage mean difference is -112.5, and the P-value is 0.212, which is statistically not significant. The LAFH mean difference is 0.8, the percentage mean difference is 1.097, and the P-value is 0.634, which is statistically not significant. The LPFH mean difference is 0.85, the percentage mean difference is 1.979, and the P-value is 0.383, which is statistically not significant. The H-ANS mean difference is -0.35, the percentage mean difference is -0.65, and the P-value is 0.505, which is statistically not significant. The H-GO mean difference is -2.55, the percentage mean difference is -10.55, and the P-value is 0.096, which is statistically not significant. Oropharyngeal dimensions as shown in Table 3.

**Table 3:** Oropharyngeal dimensions SP: Soft palate, PPA: Posterior palatal airway, PLA: Posterior lingual airway

Variables	Number of patients	Preoperative, mean±sd	Postoperative, mean±sd	Mean difference	Mean diffefrence in %	P-value
SP Length	10	32.90±5.763	32.650±6.3860	-0.25	-0.7657	0.905
SP Thickness	10	8.30±1.947	7.310000±2.09 8	-0.99	-13.54	0.117
SP Angle	10	128.40±8.40 9	127.70±11.066	-0.7	-0.5481	0.854
PPA	10	8.250±3.190 7	10.40±4.248	2.15	20.673	0.033
PLA	10	9.60±1.897	11.80±3.853	2.2	18.644	0.027

The SP length mean difference is -0.25, the percentage mean difference is -0.7657, and the P-value is 0.905, which is statistically not significant. The SP thickness mean difference is -0.99, the percentage mean difference is -13.54, and the P-value is 0.117, which is statistically not significant. The SP angle mean difference is -0.7, the percentage mean difference is -0.5481, and the P-value is 0.854, which is statistically not significant. The PPA mean difference is 2.15, the percentage mean difference is 20.673, and the P-value is 0.033, which is statistically significant. The posterior lingual airway mean difference is 2.2, the percentage mean difference is 18.644, and the P-value is 0.027, which is statistically significant.

## Discussion

Jaw irregularities may be either congenital or acquired, and orthognathic surgery seeks to address both. In addition to improving the patient’s bite and facial aesthetics, orthognathic surgery has a significant effect on the size of the patient's upper airway [7]. Orthognathic surgery is a collection of operations that may move the jaw and teeth into new positions to improve the patient's facial appearance. The back of the throat will be compressed when the jaws move [2]. The upper airway comprises the pharynx, which is a tube that runs from the nasal passages to the larynx. It is divided into three parts: the nasopharynx is the uppermost part, connected to the choanae; the oropharynx is the middle part; and the hypopharynx is the lowest part, located behind the hyoid bone. Unlike the lower airway, which is supported by bone and cartilage, the upper airway wall is entirely made of soft tissue [1].

Pharyngeal tissues such as the tonsil, sensitive sense of taste, uvula, and tongue all play an important role in mediating the size of the pharyngeal airway. The mandible and the location of the hyoid bone are two main craniofacial characteristics that determine the width of a pharyngeal airway. Any abnormality in these structures that reduces the airway's diameter might cause an obstruction [8]. The mandible, the base of the tongue, the hyoid bone, and the pharyngeal walls are all interconnected to one another through bones and ligaments. By virtue of their connections to the base of the tongue and the hyoid bone, the mandibular genioglossus, geniohyoid, and infrahyoid muscles and connective tissue attachments cause the airway to constrict and the tongue to migrate posteriorly when the jaw is retracted. Because they shape the front bulk of the pharynx, the tongue muscles play a crucial role in maintaining airway patency [9].

The position of the mandible has been shown to be connected to shifts in the hyoid bone. The hyoid bone is located farther back in Class II skeletons, where the upper airway is more constricted, and further front in Class III skeletons, where the upper airway is more open [10]. The average repositioning of the hyoid bone and persistent backward dislodging of the base of the tongue, leading to restricted pharyngeal aviation, was the anatomic modifications for OSA. Maxillomandibular advancement surgery is highly effective in treating OSA because it increases the size of the PAS and tightens the upper airway muscles and tendons by moving them closer to their bony origin [11]. Some writers have described PAS as the vertical dimension between the posterior pharyngeal wall and the base of the tongue in the mandibular plane [12].

In this research, the narrowest point between the back of the soft palate and the back of the base of the tongue was used to determine the size of the posterior airway. Unlike previous research, this one does not rely on a specific anatomically defined posterior airway area that may not even be the location of maximum constriction [3]. This study was conducted in our institution to determine the changes in the PAS for 10 patients who underwent different orthognathic surgeries. Out of the 10 patients, four underwent mandibular advancement (40%), four underwent maxillary superior impaction (40%), and one each underwent mandibular setback and maxillary rapid expansion (10%). All the patients were assessed preoperatively and postoperatively using lateral cephalograms, and the postoperative changes were calculated by subtracting the corresponding values. The collected data were statistically analyzed.

The posterior palatal airway space width preoperatively was measured as 8.25±3.19mm, and the postoperative width was 10.4±4.24mm. Thus, the percentage mean difference was calculated as 20.673. The preoperative width of the posterior lingual airway space was measured as 9.60±1.897, and the postoperative width was 11.8±3.85mm, resulting in a percentage mean difference of 18.644. These obtained values are statistically significant. There is an increase in lingual airway dimensions following mandibular advancement. It also produces a mild increase in the retropalatal airway and a change in the position of the hyoid bone and the shape of the soft palate. This is similar to the study conducted by Turnbull et al. [3], Maahs et al. [13], and Hiyama et al. [14].

When the mandible is brought forward, the intermaxillary space expands and the tongue recedes, giving the tongue greater room to move forward and creating a larger posterior lingual airway area. When the jaw advances, the soft palate changes its posture to maintain the usual contact with the dorsum of the tongue. This results in an enlarged posterior palatal airway [3]. Anterior and superior displacement of the hyoid bone is observed in our study. The preoperative vertical distance from the gonion to the hyoid bone was 26.7±11.528 and the postoperative distance was 24.150±12.1519). Therefore, the percentage mean difference is -10.55, which is not statistically significant [3].

A very mild decrease in soft palate length, soft palate thickness, and soft palate angle is noticed. The soft palate length preoperatively was 32.90±5.763, and the postoperative was 32.650±6.3860. Therefore, the percentage mean difference is -0.7657. Soft palate thickness preoperatively was 8.30±1.947, and the postoperative was 7.31±2.098. Therefore, the percentage mean difference is -13.54. The soft palate angle preoperatively was 128.40±8.409, and the postoperative was 127.70±11.066.

Following maxillary superior impaction, there is an autorotation of the mandible in a counterclockwise direction [15], that is the mandible will move forward and upward which will have a similar effect as that of mandibular advancement. This will cause an increase in PAS. This is similar to the study conducted by Alfaro et al. [5], Prado et al. [16], and Coleta et al. [17]. This research suggests that when the maxilla and mandible are rotated counterclockwise, the genial tubercles advance more than the teeth, allowing the hyoid bone, the base of the tongue, and associated soft tissues to shift forward and provide greater room in the posterior airway.

Our study observed an increase in posterior palatal airway space following surgically assisted rapid maxillary expansion. The preoperatively posterior palatal airway was 8mm, and the postoperative airway was 10mm. However, the condition of the posterior lingual airway did not alter. Maxillary enlargement may cause a reduction in nasal resistance and an increase in posterior palatal airway space due to the ensuing enlargement of the nasal floor and nasal cavity. This is consistent with the findings of research by Bannink et al. [18], which found that posterior tongue and oral tissue displacement may be avoided when the maxilla is expanded in a transverse direction.

According to the study conducted by Lye et al. [2], The anterior, posterior, and lateral dimensions of the airway were not noticeably widened by maxillomandibular expansion. Only one patient underwent a mandibular setback in this study. Following that, an increase in the posterior palatal airway and a decrease in the posterior lingual airway were noticed. That is, the posterior palatal airway preoperatively was 6mm and the posterior palatal airway postoperatively was 8mm. The preoperative posterior lingual airway was 10mm and the post-operative lingual airway was 8mm. This is similar to the study conducted by Samman et al. [19] and Pereira-Filho et al. [20], which show a decrease in the posterior lingual airway and a compensatory increase in the nasopharyngeal and oropharyngeal airway. According to the study conducted by Turnbull et al. [3] and Hasebe [4]. There is a decrease in both posterior lingual airway space and posterior palatal airway space. The vertical distance of the hyoid bone preoperatively was 19mm, and post-operative was 22.5mm, indicating the downward displacement of the hyoid bone. Similar changes were observed by Turnbull et al. [3]. We noticed that there is a change in posterior palatal airway space and posterior lingual airway space as a result of orthognathic surgery.

The study has some limitations, such as the need for further research with larger samples to establish a firm conclusion. Although barium sulphate paste was used to enhance the visibility of the posterior pharyngeal airway in lateral cephalograms, it may not accurately represent the dynamic changes and dimensions of the airway during different functional conditions, such as breathing or swallowing. The study focused primarily on the anatomical changes in the posterior pharyngeal airway following orthognathic surgery. The impact of these changes on functional outcomes, such as breathing and sleep quality, was not assessed. It was a short-term period of the study only so more duration has to be taken for further studies.

## Conclusions

It is essential to consider these soft tissue changes when planning orthognathic procedures, as alterations in the pharyngeal airway may impact the patient's postoperative breathing and overall health. Patients with OSA or those at risk of developing it should be closely evaluated and managed appropriately during the surgical planning process. Overall, this study provides valuable insights into the potential effects of orthognathic surgery on the posterior pharyngeal airway, contributing to the ongoing advancement and refinement of orthognathic surgical techniques for better patient outcomes. Further research in this area is warranted to deepen our understanding of these interactions and optimize treatment approaches for patients with OSA and related conditions.
